# Cross-sectional and longitudinal associations between 24-hour movement behaviors and growth, motor, and social-emotional development in early childhood

**DOI:** 10.1186/s44167-025-00085-9

**Published:** 2025-08-28

**Authors:** Jelle Arts, Teatske M. Altenburg, Annelinde Lettink, Arnoud P. Verhoeff, Jessica S. Gubbels, Mai J. M. Chinapaw

**Affiliations:** 1 Amsterdam UMC location Vrije Universiteit Amsterdam, Public and Occupational Health, De Boelelaan 1117, Amsterdam, The Netherlands; 2 Amsterdam Public Health, Health Behaviors & Chronic Diseases, Amsterdam, The Netherlands; 3 Amsterdam Public Health, Methodology, Amsterdam, The Netherlands; 4 Public Health Service Amsterdam, Sarphati Amsterdam, Amsterdam, 1018 WT The Netherlands; 5 Department of Sociology, University of Amsterdam, Amsterdam, 1018 WV The Netherlands; 6 Department of Health Promotion, Maastricht University, NUTRIM Institute of Nutrition and Translational Research in Metabolism, PO Box 616, Maastricht, 6200 MD The Netherlands

**Keywords:** Infant, Toddler, Physical activity, Sedentary behavior, Sleep, Body mass index, Development, Compositional data analysis

## Abstract

**Background:**

To enhance evidence on optimal 24-hour movement behaviors (physical activity, sedentary behavior, and sleep) in early childhood, this study investigated cross-sectional and longitudinal associations of the composition of these behaviors with social-emotional development, gross motor development and growth in 0–4-year-olds.

**Methods:**

Data were collected at two timepoints (baseline and 9 months later) in two sub-cohorts from the My Little Moves study: one examining social-emotional development (sub-cohort-SE) and one gross motor development and growth (sub-cohort-GM). Children’s time spent in 24-hour movement behaviors was assessed via parent-report using the My Little Moves app. Isometric log-ratios were calculated to represent 24-hour movement behavior composition. Social-emotional and gross motor development were assessed using the Bayley Scales of Infant and Toddler Development-III, with both total raw and norm-referenced scaled scores. Children’s weight and height were measured to calculate BMI z-scores. Linear regression and mixed-model analyses examined cross-sectional and longitudinal associations, with significant results further explored using compositional isotemporal reallocation analysis.

**Results:**

Sub-cohort-SE provided data from 101 children at timepoint 1 (age 20.6 ± 12.5 months) and 62 children at timepoint 2 (age 25.7 ± 9.8 months). Sub-cohort-GM provided data from 60 children at timepoint 1 (age 20.4 ± 10.8 months) and 46 children at timepoint 2 (age 27.6 ± 9.6 months). The composition of 24-hour movement behaviors was significantly associated with raw gross motor development scores in both cross-sectional (*p* < .001, *R²Δ* = 0.042) and longitudinal (*p* < .001, *R²Δ* = 0.033) analyses. The association with BMI z-scores was significant only in the cross-sectional analysis (*p* = .015, *R²Δ* = 0.130). Reallocating 10 min from sedentary behavior to physical activity or sleep increased raw gross motor development scores by 0.22 (95% CI [0.11, 0.33]), and 0.27 (95% CI [0.08, 0.45]). Reallocating 10 min from sedentary behavior to sleep increased BMI z-scores by 0.04 (95% CI [0.01, 0.06]).

**Conclusions:**

The composition of 24-hour movement behaviors was significantly associated with BMI z-scores and gross motor development, but not social-emotional development in children aged 0–4 years. Evidence on the optimal distribution of movement behaviors remains unclear and needs further examination in larger longitudinal studies.

**Supplementary Information:**

The online version contains supplementary material available at 10.1186/s44167-025-00085-9.

## Introduction

Early childhood (0–5 years) represents a crucial developmental phase in life, characterized by rapid changes in physical, cognitive, and social-emotional domains [1]. During this period, a significant portion of the brain’s structure and capacity is shaped, making it also a sensitive phase for a healthy development [1–3]. Consequently, these formative years lay the foundation for a child’s lifelong health and well-being [1, 2]. For example, social-emotional development in early childhood has an important role in forming healthy relationships, supporting mental health, and promoting overall well-being later in life [4]. Additionally, gross motor development is not only essential for physical health but also serves as a predictor of later psychosocial challenges, cognitive development, and academic achievement [5–8]. Moreover, deviations from healthy BMI in young children may indicate long-term health problems such as obesity and metabolic disorders [9, 10]. As such, ensuring that children’s experiences during the early years support their healthy growth and development is of utmost importance [11].

In the last decade, the role of 24-hour movement behaviors, encompassing physical activity (PA), sedentary behavior (SB), and sleep, on young children’s development has gained increased attention [12, 13]. To support the healthy growth and development of young children, the World Health Organization (WHO) has formulated 24-hour movement guidelines tailored to different age subgroups: infants (0–1 year), toddlers (1–3 years), and preschoolers (3–5 years) [14, 15]. These guidelines, offering recommendations for time spent in PA, SB, and sleep, serve as valuable tools for parents, caregivers, healthcare and childcare professionals, and policymakers to support optimal 24-hour movement behaviors in young children.

Despite these guidelines being grounded on the best available evidence, concerns persist regarding the quantity and quality of the evidence, which is considered low [14, 16]. An important reason for this is the inconsistency in evidence on associations between PA [11, 17], SB [18], sleep [19], and the combination of these 24-hour movement behaviors [12, 20, 21] with health and development in early childhood. Consequently, the current guidelines are largely based on expert opinion [14, 16]. One possible explanation for the lack of evidence is that most studies examining associations between 24-hour movement behaviors and young children’s healthy growth and development have used a cross-sectional design [11, 12, 17–21]. Furthermore, since these studies predominantly focused on preschoolers, current evidence is especially limited for infants and toddlers [22]. For example, a systematic review by Rollo et al. (2020) on the association between all 24-hour movement behaviors and several health indicators (including growth and development) identified only one study in infants and two in toddlers, while ten studies in preschoolers were identified [12].

To address this gap, Carson et al. (2022) studied longitudinal associations between 24-hour movement behaviors and development in infants. They examined associations of PA, SB, and sleep separately, and found that tummy time and reading time were associated with motor and personal-social development [23]. However, considering that different movement behaviors are co-dependent and mutually exclusive, there is a compelling argument for examining the composition of 24-hour movement behaviors instead of each behavior separately, for example by using compositional data analysis [21, 24, 25]. Unfortunately, so far only a few studies have examined the relationship between the composition of 24-hour movement behaviors and young children’s healthy growth and development, of which only one included children under the age of three [21]. Therefore, there is an urgent need for studies examining this relationship in infants and toddlers [22].

This study aimed to bridge this gap by investigating both the cross-sectional and longitudinal associations of the composition of 24-hour movement behaviors with BMI z-scores, social-emotional, and gross motor development of children aged 0–4 years. By addressing this, we aimed to provide valuable insights that can inform evidence-based recommendations for promoting optimal 24-hour movement behaviors in early childhood, thereby stimulating children’s healthy growth and development. We hypothesized that the composition of 24-hour movement behaviors would be associated with social-emotional development, gross motor development, and BMI z-scores. Specifically, in both cross-sectional and longitudinal associations, we expected both PA and sleep, relative to the other behaviors, to be positively associated with social-emotional and gross motor development, and negatively associated with BMI z-scores. Conversely, we expected SB, relative to the other behaviors, to be negatively associated with social-emotional and gross motor development, and positively associated with BMI z-scores.

## Methods

### Design

This study is part of “My Little Moves”, a longitudinal observational cohort study conducted in the Netherlands. The My Little Moves study was divided into three sub-cohorts, each focusing on different research questions, to limit the burden for participants. A comprehensive outline of the My Little Moves study protocol is available elsewhere [26]. For the current study, we used data from two timepoints (baseline and approximately 9 months later) from two sub-cohorts: one focusing on young children’s 24-hour movement behaviors and social-emotional development (sub-cohort-SE) and another focusing on young children’s 24-hour movement behaviors, growth, and gross motor development (sub-cohort-GM). This study was reported in accordance with the STROBE guidelines for observational studies (Additional file 1) [27].

### Participants and recruitment

We aimed to include 200 parents and their children in both sub-cohorts. Children were eligible if they met the following criteria: (1) aged 0–4 years (up to 48 months), (2) born > 32 weeks of gestation, and (3) without parent-reported developmental disorders or medical diagnoses that might influence their 24-hour movement behaviors or development. Additionally, parents of included children needed to be able to read the Dutch language and own a smartphone or tablet device.

For both sub-cohorts, we recruited children and their parents through early childhood education and care (ECEC) services (e.g., daycares) and youth health care services. Parents received information about the study through email, newsletters and/or flyers. For sub-cohort-SE, parents and children were additionally recruited through community organisations such as sports clubs, and at public spaces such as playgrounds. Moreover, we recruited parents and children from the dynamic Sarphati Cohort (https://www.sarphaticohort.nl/en/), which includes children aged 0–4 years in Amsterdam [28]. Parents of children in the Sarphati Cohort received an information letter via email from Sarphati. Informed consent was obtained from all parents before their participation in the study, using survey software Survalyzer (Survalyzer BV, Utrecht, the Netherlands) or a paper form. The study protocol received approval from the Medical Ethics Committee of the Amsterdam University Medical Centres (Protocol No. 2022.0020).

### Procedures

Data on 24-hour movement behaviors, social-emotional development, gross motor development, and growth were collected at both timepoints. In sub-cohort-SE, data were collected between May 2022 and August 2023 for timepoint 1 and between March 2023 and April 2024 for timepoint 2. In sub-cohort-GM, data were collected between October 2022 and September 2023 for timepoint 1 and between July 2023 and April 2024 for timepoint 2. After consenting to participate, parents reported their child’s date of birth and sex through the consent form. Child age at each timepoint was calculated from the date of birth. Following this, parents provided their own age, gender, highest level of education (categorized as lower, medium and high according to the International Standard Classification of Education [29]), and the country of birth of the child’s mother and father. For parents recruited through the Sarphati Cohort, this information was obtained from data previously collected within the cohort. For all other parents, a brief online survey was conducted using Castor Electronic Data Capturing software (Castor EDC, Amsterdam, The Netherlands).

Subsequently, data on 24-hour movement behaviors were collected by parent-report using a mobile application (app) developed for the current cohort study, named the My Little Moves app [30]. Parents received an email in which they were asked to complete the My Little Moves app for seven consecutive days. This email also contained detailed guidance on how to download and complete the app. Data on children’s social-emotional development were collected through an online questionnaire sent through Castor EDC. Children’s gross motor skills and growth were assessed by two researchers (JA, AL, or trained research assistants) at the ECEC service or at home, in consultation with parents.

### 24-hour movement behaviors

#### My little moves app

The development of the My Little Moves app has previously been described in detail [30]. The app has a time-use diary format through which parents report the daily activities of their child, using the following activity categories: (1) personal care, (2) eating/drinking, (3) sitting/lying calmly, (4) screen use, (5) passive transport, (6) active transport, (7) playing, (8) sleeping, (9) other activity, (10) I don’t know, and 11) my child was with someone else. Depending on the selected activity, parents are prompted to provide additional details regarding the intensity (e.g., active or calm play), posture (e.g., lying on tummy or back) and/or context (e.g., location) of the activity. Before completing the time-use diary, parents provide their child’s age (0–6 months, 6–12 months, 1–2 years, 2–3 years, and 3–4 years) and achieved motor milestones in the app (i.e., depending on the child’s age: roll over from back to belly, roll over from belly to back, sit without support, crawl, stand without support, walk without support). This information enables the app to tailor its content, including activity categories and follow-up questions, to the child’s developmental stage.

In a content validity study, the My Little Moves app was considered sufficiently relevant, comprehensive, comprehensible and user-friendly [30]. Parents typically spent between 10 and 30 min (min) per day reporting their child’s activities in the app. A comparative analysis with accelerometer data provided preliminary evidence for the app’s ability to assess 24-hour movement behaviors of young children, with reliable results provided when activities are reported for at least two full days [31].

#### Data preprocessing

Using customized R-scripts, we calculated the daily duration of time spent in each activity category (in min) for each participant. Subsequently, we calculated the duration of PA, SB and sleep (total duration, regardless of whether it occurred during the day or night) from the developmentally appropriate activity categories and follow-up questions on intensity and posture. The classification of these 24-hour movement behaviors from the activity categories and follow-up questions in the app is detailed elsewhere [31]. For inclusion in the final analysis, parents were required to report their child’s activities for a minimum of two days, with each day comprising at least 23 hours, excluding the categories ‘other activity,’ ‘child was with someone else’ and ‘I don’t know’ [31]. For each participant with valid data, the mean daily time spent in PA, SB, and sleep was calculated and used in analysis.

### Social-emotional and gross motor development

We assessed children’s social-emotional development using the Social Emotional Scale of the Dutch version of the Bayley Scales of Infant and Toddler Development-Third Edition (Bayley-III-NL) [32, 33]. This parent-reported scale assesses the functional emotional milestones achieved by young children. Prior research has demonstrated the scale’s reliability and discriminant validity [32, 33]. The scale contains 11 items for infants aged 0–3 months, and items are added as the child gets older, reaching a maximum of 35 items. Each item is scored on a 5-point scale: 1 = none of the time; 2 = some of the time; 3 = half of the time; 4 = most of the time; and 5 = all of the time, or can’t tell. Subsequently, item scores were summed to calculate the total raw score, representing social-emotional development.

We used the Gross Motor Scale of the Bayley-III-NL to assess children’s gross motor development, including a total of 72 items. Previous studies showed sufficient reliability, convergent validity, and discriminant validity of this sub-scale [32–34]. A score of ‘1’ was assigned when a child successfully completed an item, while a score of ‘0’ was given for unsuccessful attempts. If a child scored ‘0’ on any of the first five items, testing resumed at the entry point corresponding to the previous age level. The test finished when a child scored ‘0’ on five consecutive items. Items from earlier age levels that were not administered were automatically scored positive (score 1). To calculate the total raw score, we summed the number of points obtained.

Additionally, for both social-emotional and gross motor development, we converted the total raw scores into norm-referenced scores based on Dutch reference values using the online platform Q-Global (Pearson Benelux B.V., Amsterdam, the Netherlands). Specifically, scaled scores ranging from 1 to 19 were used, with a score of 10 indicating average development. Since reference values for the Bayley-III-NL are only available for children aged from 16 days to 42 months and 15 days, scaled scores could not be calculated for children outside this age range. It is important to note that in the current study, social-emotional and gross motor development outcomes are presented as static indicators at each timepoint.

### Growth

Trained researchers measured children’s weight (kg) and height (cm) to compute the Body Mass Index (BMI) z-score. Weight was measured to the nearest 0.1 kg using a calibrated electronic (baby) scale (Seca 354). Height was measured to the nearest 0.1 cm using a portable stadiometer (Marsden HM-250P) or a length board (Seca 417) depending on the child’s ability to stand. Children were lightly dressed and barefoot during these measurements. Both weight and height measurements were conducted twice, and if the second measurement deviated by more than 0.2–0.5 cm from the first, a third measurement was conducted. The average of the two or three measurements was taken [35]. Finally, based on the children’s height, weight, age and sex, we used R package Anthro developed by the WHO to compute BMI z-scores [36].

### Analyses

Statistical analyses were conducted using SPSS version 28 for initial data exploration and to characterize the study population, while all subsequent analyses were performed in RStudio version 4.3.2. Statistical significance was set at alpha = .05. First, recognizing that time spent in each 24-hour movement behavior is mutually exclusive, we transformed the time allocated to PA, SB, and sleep into compositional variables using the isometric log-ratio (ilr) method [18, 19, 23]. We calculated three sets of two ilr-coordinates that together represented the composition of 24-hour movement behaviors using the compositions package in R [37]. In each set, the first ilr-coordinate (ilr1) represented one reference behavior (PA, SB, or sleep) relative to the remaining behaviors, while the second ilr-coordinate (ilr2) represented the remaining behaviors relative to each other.

To identify potentially unlikely measurements, we examined outliers, defined as z-scores outside the range of −3 to 3 for ilr-coordinates, social-emotional development (raw and scaled), gross motor development (raw and scaled), or BMI z-scores. A total of eight outliers were removed: six for ilr-coordinates, one for social-emotional development, and one for BMI z-score. Following outlier removal, we calculated the baseline characteristics of all participants included in the analyses. To examine potential differences between the two sub-cohorts, an independent samples t-test was conducted to assess differences in mean age, and a chi-square test was used to assess differences in sex distribution. Additionally, for time spent in PA, SB, and sleep; social-emotional development scores; gross motor development scores; and BMI z-scores at both timepoints, we calculated means and standard deviations for normally distributed data, or medians and interquartile ranges when variables were not normally distributed at any timepoint.

Cross-sectional associations were examined using linear regression analyses, with the lm function in R. For each child, data from the first timepoint were used when available; otherwise data from the second timepoint were used. Ilr1 and ilr2 were included as independent variables, with social-emotional development scores (raw or scaled), gross motor development scores (raw or scaled), or BMI z-scores as dependent variables, respectively. Age and sex of the child were included as covariates in all regression models. We checked the assumptions for linear regression through visual inspection of residual plots. The normality assumption was not met for the raw social-emotional development and raw gross motor development scores. Despite several transformations, the residuals still deviated from a normal distribution. Consequently, for these outcomes, measurements with a Cook’s distance greater than 4/total number of observations and a studentized residual outside the range of −2 to 2 were excluded (four measurements for social-emotional development, and three for gross motor development) [38]. A sensitivity analysis was conducted to assess whether removing these measurements affected our findings, and if so, this is indicated in the tables.

We analyzed longitudinal associations using linear mixed-effects models with the lme function from the nlme package in R, incorporating ilr1, ilr2, the child’s sex and age as fixed effects, and including a random intercept for participant number to account for within-subject correlation due to repeated measurements [39]. Again, the dependent variables were social-emotional development scores (raw or scaled), gross motor development scores (raw or scaled), or BMI z-scores. Using data from both timepoints, these models assessed whether differences in movement behavior compositions were associated with differences in these outcomes over time. Linear mixed models use maximum likelihood estimation to handle missing data, eliminating the need for data imputation [40, 41]. Consequently, participants were included in the models if they had complete data for each independent and dependent variable at least at one timepoint. A sensitivity analysis was conducted to compare associations among participants with complete data at both timepoints against the main analysis, which included participants with data from at least one timepoint. As with the cross-sectional data, the residuals for the raw social-emotional and gross motor development scores were not normally distributed, necessitating similar removal of measurements based on Cook’s distance and studentized residual values (three for both social-emotional development and gross motor development).

For both linear regression and mixed model analyses, we compared the full model (covariates and ilr-coordinates) with a null model (covariates only), using the log-likelihood ratio. Specifically, ANOVA tables were examined to assess whether adding ilr1 and ilr2 significantly improved the model fit, thereby indicating an association between movement behavior composition and the dependent variable. Additionally, to explore the contribution of each 24-hour movement behavior, we ran three models, each using a different set of ilr-coordinates. For each model, we focused on the regression coefficient (including the 95% confidence interval (CI) and p-value) of the first coordinate (ilr1) to determine which behavior was most strongly associated with the dependent variable. The explained variance (R-squared change values) attributed to the set of ilr-coordinates were interpreted according to Cohen’s guidelines: very small (.00–.02), small (.02–.13), medium (.13–.26), and large (> .26) [42].

For all growth and development outcomes that were significantly associated with movement behavior composition, we conducted compositional isotemporal reallocation analyses [43]. These analyses help to interpret the observed associations, and estimate how reallocating a fixed duration of time between movement behaviors would affect these outcomes. To the best of our knowledge, this method has not yet been implemented in longitudinal mixed-model analysis, and appropriate R packages are currently unavailable. As developing such methodology was beyond the scope of the present study, we only applied the compositional isotemporal reallocation analyses to the cross-sectional data. We used the predict_delta_comps function from the codaredistlm R package to create the reallocation models [44]. Both proportional reallocation and one-by-one reallocation models were applied in 10 min-increments ranging from − 60 to 60 min. In proportional reallocation, time is proportionally added to or taken from one behavior and distributed among the other behaviors (e.g., reducing PA by 10 min while increasing SB and sleep by 5 min each). In one-versus-one reallocation, time is shifted directly between two behaviors while holding the third constant (e.g., reducing PA by 10 min and increasing SB by 10 min, with sleep unchanged). We chose 10-min increments because this duration seems feasible for implementation in real-life settings and aligns with previous research in early childhood [45, 46]. The reallocation models calculated predicted changes in growth and development scores by subtracting the predicted values derived from the new compositions from those derived from the initial composition (i.e., ilr1 and ilr2). Predicted changes were considered statistically significant if the 95% CIs of these changes excluded zero. Our R scripts for creating the ilr-coordinates, as well as for conducting the linear regression, linear mixed model, and compositional isotemporal reallocation analyses, are available in Additional file 2.

## Results

### Descriptives

**Fig. 1:**
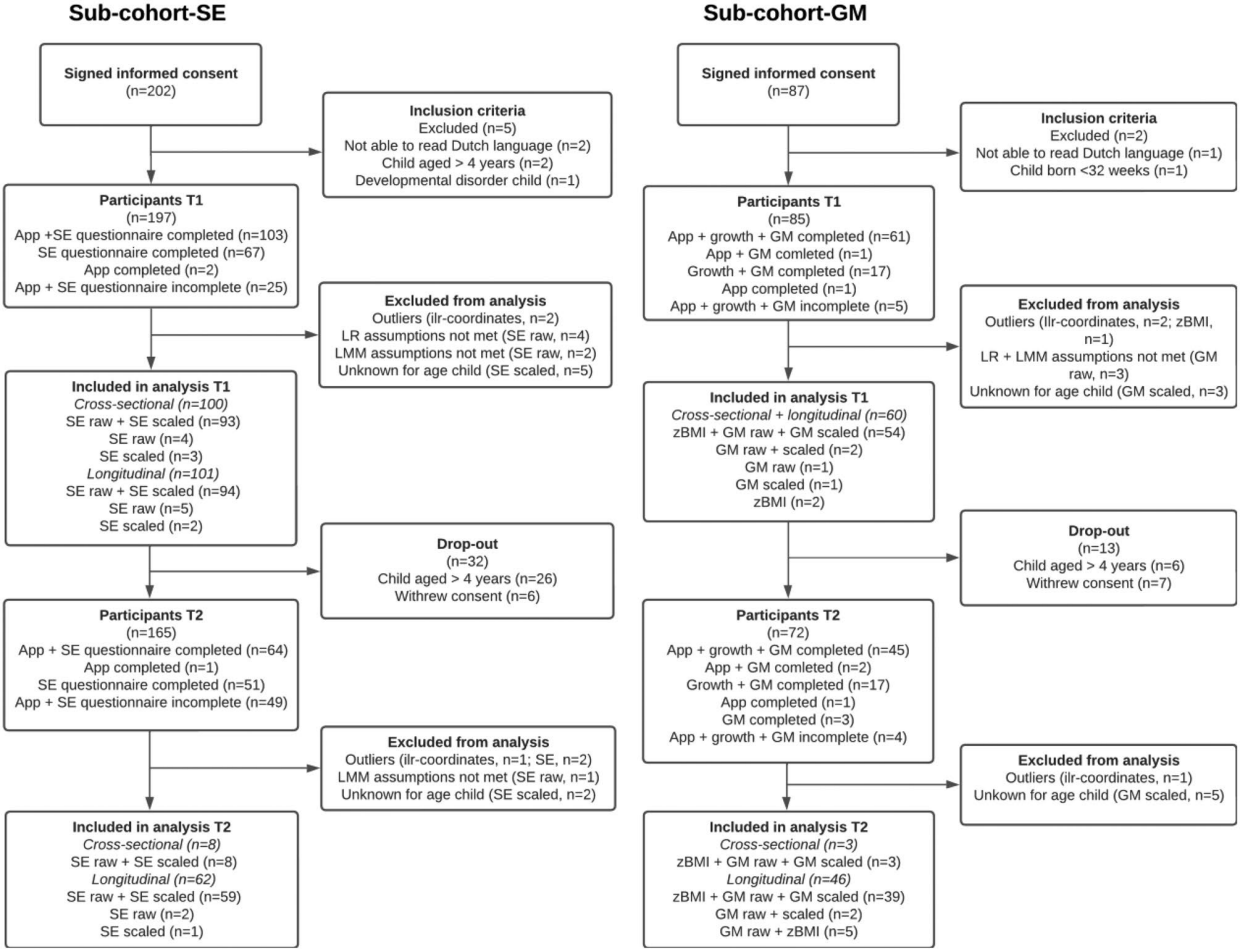
Participant flowcharts for sub-cohort-SE (social-emotional development) and sub-cohort-GM (gross motor development and growth) at timepoint 1 (T1, baseline) and timepoint 2 (T2, 9-months follow-up). The flowcharts specify which social-emotional development scores (SE raw and SE scaled), gross motor development scores (GM raw and GM scaled), and BMI z-scores (zBMI) were included in both linear regression (LR) and linear mixed model (LMM) analyses

Figure 1 presents the participant flowcharts for sub-cohort-SE and sub-cohort-GM. In sub-cohort-SE, 202 parents initially signed consent for participation, with 197 parents and children meeting the inclusion criteria. After removing outliers, complete data were available for 101 children at the first timepoint (mean age 20.6 ± 12.5 months, 45.5% girls), and 62 children at the second timepoint (mean age 25.7 ± 9.8 months, 48.4% girls). In sub-cohort-GM, 87 parents signed up, with 85 parents and children meeting the inclusion criteria. After removing outliers, complete data were available for 60 children at the first timepoint (mean age 20.4 ± 10.8 months, 43.3% girls), and 46 children at the second timepoint (mean age 27.6 ± 96 months, 45.6% girls). There were no significant differences between children in sub-cohort-SE and sub-cohort-GM in terms of mean age (*p* = .49) or sex distribution (*p* = .97). The mean interval between timepoints was 8.7 months (range = 7.5–9.7 months) in sub-cohort-SE and 9.3 months (range = 6.9–11.8 months) in sub-cohort-GM. In total, 32 children were not followed-up at the second timepoint because they had turned 4 years old. Additionally, scaled scores could not be computed for seven children in sub-cohort-SE and eight children in sub-cohort-GM because no norm-referenced scoring was available. Table 1 provides an overview of the characteristics of participating children and their parents at the first timepoint. The majority of participating parents were female (94.1% in sub-cohort-SE and 86.4% in sub-cohort-GM) and highly educated (94.7% in sub-cohort-SE and 85.7% in sub-cohort-GM).

Table 2 presents descriptive data on 24-hour movement behaviors, growth, and developmental outcomes of children at both timepoints. For children in sub-cohort-SE, the proportions of time spent on PA, SB, and sleep were 15.7%, 28.4%, and 55.9%, respectively, at timepoint 1. At timepoint 2 these proportions were 14.3%, 30.3%, and 55.4%, respectively. Similarly, in sub-cohort-GM, proportions of time spent on PA, SB, and sleep were 14.2%, 30.2%, and 55.6%, respectively, at timepoint 1, and 13.2%, 32.3%, and 54.5%, respectively, at timepoint 2. The median scaled scores for social-emotional development and the mean scaled scores for gross motor development ranged from 9 to 10, indicating that most children were developing at an average level.

**Table 1:** Characteristics of children and their parents at the first timepoint in sub-cohort-SE (social-emotional development) and sub-cohort-GM (gross motor development and growth)

	Sub-cohort-SE	Sub-cohort-GM
**Number of children** (No. 0–1 yrs / 1–2 yrs / 2–3 yrs / 3–4 yrs)	101 (33 / 27 / 28 / 13)	60 (14 / 25 / 15 / 6)
**Age child in months** (Mean (SD), range)	20.6 (12.5), 3.0–48.5	20.4 (10.8), 3.8–46.7
**Sex child** (No. Boy/Girl)	55 / 46	34 / 26
**Age parent in years** (mean (SD), range)	36.0 (3.5),29.3–45.6 *	35.5 (5.3), 32.9–59.8
**Gender of reporting parent** (No. Male / Female)	6 / 95	8 / 52
**Level of education of reporting parent** (No. Lower / Medium / High)	2 / 2 / 71 *	0 / 8 / 48 **
**Country of birth mother child** (No. Netherlands / Other)	71 / 13 *	43 / 13 **
**Country of birth father child** (No. Netherlands / Other)	68 / 11 *	44 / 12 **

* *N* = 21, 25, 17, and 22 missings for age parent, level of education, country of birth mother, and country of birth father, respectively;

** *N* = 4 missings for level of education, country of birth mother, and country of birth father

**Table 2:** Time spent on 24-hour-movement behaviors, developmental scores, and growth of children in sub-cohort-SE (social-emotional development) and sub-cohort-GM (growth and gross motor development) at both timepoints. Depending on the distribution, values are presented as mean ± standard deviation or median [Interquartile range]

	Sub-cohort-SE		Sub-cohort-GM	
	Timepoint 1	Timepoint 2	Timepoint 1	Timepoint 2
PA (min/day)	224.3 ± 81.1	205.1 ± 84.6	186.8 [137.2–264.2]	176.0 [120.2–221.6]
SB (min/day)	401.0 [338.7–460.0]	410.0 [364.4–495.6]	432.6 ± 117.9	463.7 ± 113.5
Sleep (min/day)	807.0 [740.8–867.5]	787.3 [728.1–858.1]	795.0 ± 80.1	781.2 ± 90.4
SE raw score *	99.0 [68.0–136.0]	112.0 [84.5–139.5]	-	-
SE scaled score *	10.0 [8.0–12.0]	9.0 [7.0–11.0]	-	-
GM raw score *	-	-	49.0 [39.0–55.0]	58.0 [53.0–62.3]
GM scaled score *	-	-	9.5 ± 3.3	9.8 ± 3.0
BMI z-score	-	-	0.7 ± 0.8	1.0 ± 0.9

Abbreviations: BMI body mass index, GM gross motor development, PA physical activity, SB sedentary behavior, SE social-emotional development

* Developmental scores were based on the Social Emotional Scale and Gross Motor Scale of the Bayley-III-NL. Raw scores reflect total scores, with GM scores ranging from 0 to 72, and SE scores ranging from 0 to a maximum of 11 to 35, depending on the child’s age. Scaled scores are norm-referenced, ranging from 1 to 19 [32, 33]

### Compositional analyses

Tables 3 and 4 present the cross-sectional and longitudinal associations between the composition of 24-hour movement behaviors and social-emotional development, gross motor development, and BMI z-scores. We found a significant association between movement behavior composition and raw gross motor development scores in both the cross-sectional and longitudinal analyses (*p* < .001). However, the explained variances of the ilr-coordinates on these scores were small (*R²Δ* = .042 in the cross-sectional and *R²Δ* = .033 in the longitudinal analyses). Additionally, a significant association was found between movement behavior composition and BMI z-scores only in the cross-sectional analysis (*p* = .015), with a medium explained variance (*R²Δ* = .130). No significant associations were observed between movement behavior composition and the scaled gross motor development and both scaled and raw social-emotional development scores (.060 ≥ *p* ≤ .986).

When examining the first ilr-coordinates, more SB, relative to PA and sleep, was associated with lower raw gross motor development scores in both the cross-sectional (*β* = −9.43, *p* = .002) and longitudinal analyses (*β* = −11.27, *p* < .001). In contrast, more sleep, relative to the other behaviors, was associated with higher raw gross motor development scores in both the cross-sectional (*β* = 8.51, *p* = .029) and longitudinal analyses (*β* = 13.06, *p* < .001). More SB, relative to the other behaviors, was associated with lower BMI z-scores (*β* = −1.28, *p* = .005 in the cross-sectional, and *β* = −0.91, *p* = .027 in the longitudinal analyses), while more sleep, relative to the other behaviors, was associated with higher BMI z-scores (*β* = 1.46, *p* = .016 in the cross-sectional, and *β* = 1.20, *p* = .026 in the longitudinal analyses).

### Compositional isotemporal reallocation analyses

**Fig. 2:**
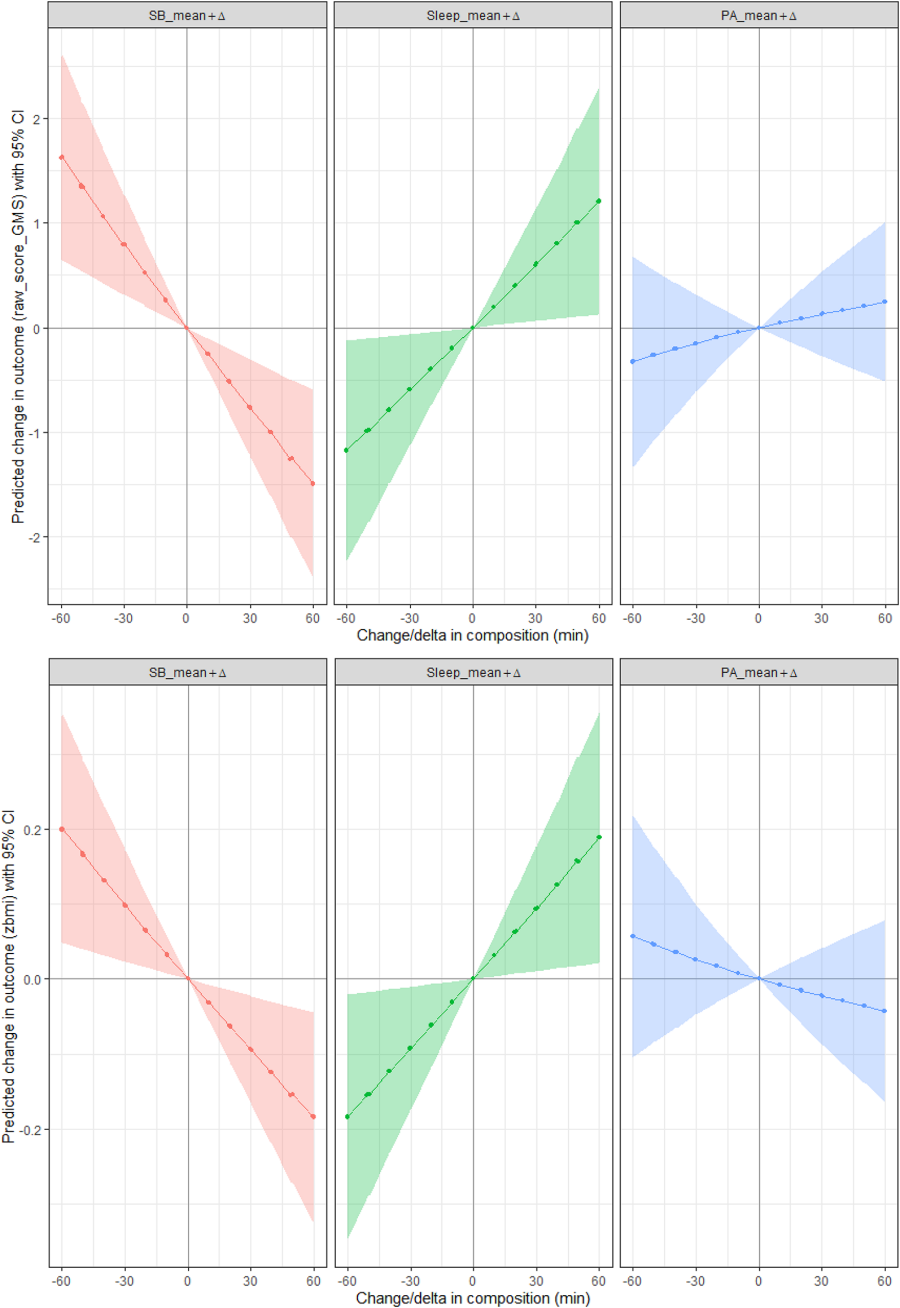
Prediction of raw gross motor scores (upper graphs) and BMI z-scores (lower graphs) through proportional reallocation of time spent in 24-hour movement behaviors. The prediction models show changes in scores when reallocating time to one behavior equally from the other behaviors in steps of 10 min over a time frame of −60 to 60 min. The shaded zone on each graph shows the 95% CI

**Fig. 3:**
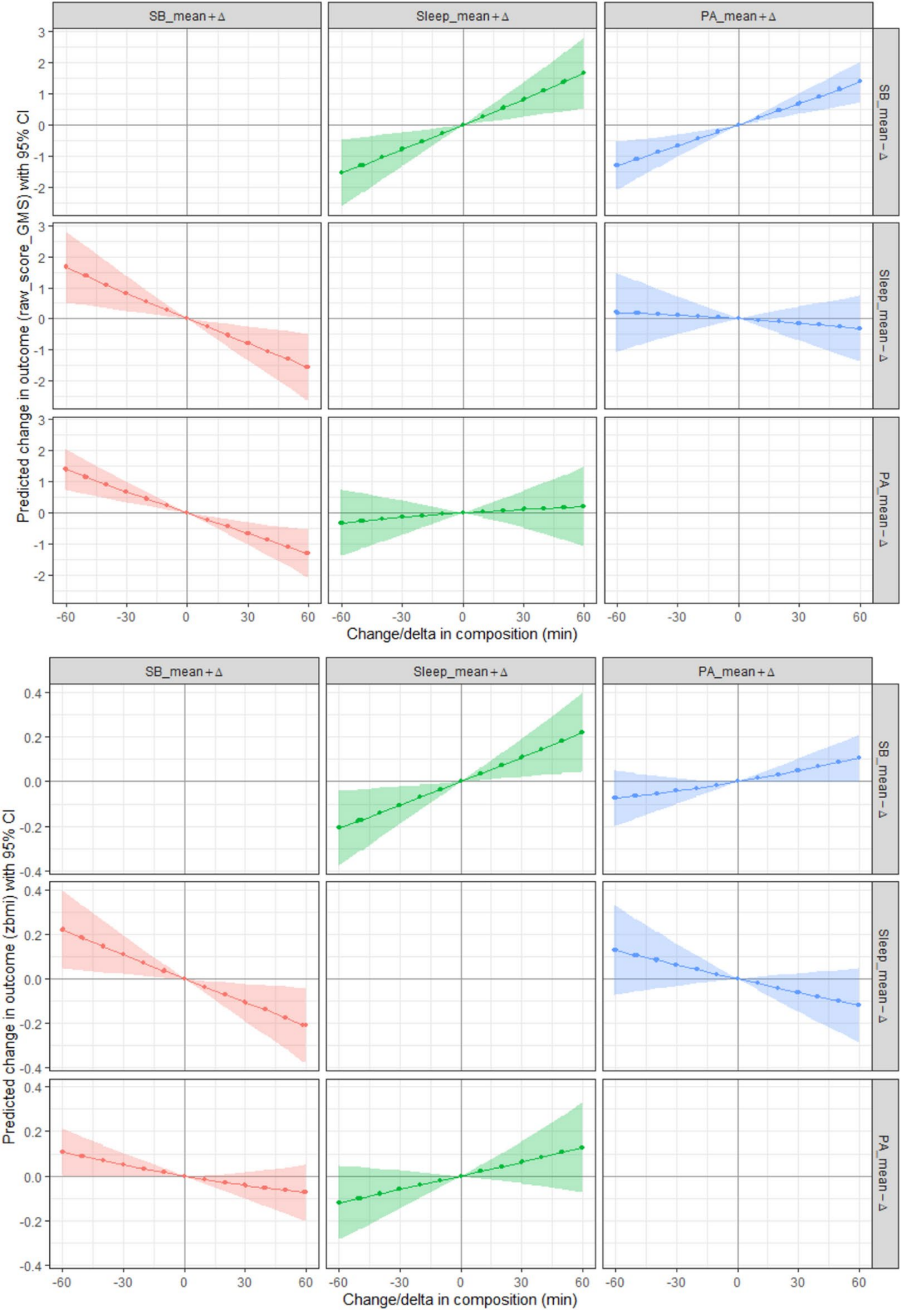
Prediction of raw gross motor scores (upper graphs) and BMI z-scores (lower graphs) through one-by-one reallocation of time spent in 24-hour movement behaviors. The prediction models show changes in scores when reallocating time from one behavior (right vertical axis) to another behavior (upper horizontal axis) in steps of 10 min over a time frame of − 60 to 60 min. The shaded zone on each graph shows the 95% CI

Figures 2 and 3 illustrate the predicted changes in raw gross motor development scores and BMI z-scores following proportional reallocation of time spent in 24-hour movement behaviors, and one-by-one reallocation of time spent in 24-hour movement behaviors, respectively. An additional 10 min in SB, proportionally taken from PA and sleep, was significantly associated with a decrease in raw gross motor development scores (−0.26; 95% CI [−0.42, − 0.10]), while reallocating 10 min from SB to PA and sleep resulted in significantly higher raw gross motor development scores (0.26; 95% CI [0.10, 0.41]). Additionally, 10 min more sleep, proportionally taken from PA and SB, resulted in a significant increase raw gross motor development scores (0.20; 95% CI [0.02, 0.37]), while reallocating 10 min of sleep to PA and SB was associated with a decrease in raw gross motor development scores (−0.20; 95% CI [−0.37, −0.02]). Proportionally reallocating time from or to PA did not significantly change raw gross motor development scores (−0.05; 95% CI [−0.19, 0.10], and 0.05; 95% CI [−0.09, 0.18], respectively). For BMI z-scores, an additional 10 min in SB, proportionally taken from PA and sleep, was significantly associated with a decrease (−0.03; 95% CI [−0.06, −0.01]), while reallocating 10 min from SB to PA and sleep resulted in significantly increased BMI z-scores (0.03; 95% CI [0.01, 0.06]). Additionally, 10 min more sleep, proportionally taken from PA and SB, resulted in a significant increase in BMI z-scores (0.03; 95% CI [0.004, 0.06]), while reallocating 10 min of sleep to PA and SB was associated with a decrease in BMI z-scores (−0.03; 95% CI [−0.06, 0.004]). Proportionally reallocating time from or to PA did not significantly change BMI z-scores (0.01; 95% CI [−0.01, 0.03], and −0.01; 95% CI [−0.03, 0.01], respectively).

Table 5 presents the predicted changes in raw gross motor development scores and BMI z-scores following 10-min one-by-one reallocations of time spent in 24-hour movement behaviors. Reallocating 10 min from sleep to SB resulted in a significant decrease in raw gross motor development scores (−0.26; 95% CI [−0.45, −0.08]), while adding 10 min of sleep by reducing SB significantly increased raw gross motor development scores (0.27; 95% CI [0.08, 0.45]). In addition, reallocating 10 min from SB to PA significantly increased raw gross motor development scores (0.22; 95% CI [0.11, 0.33]), whereas allocating 10 min from PA to SB resulted in a significant decrease in gross motor development scores (−0.22; 95% CI [−0.33, −0.11]). Furthermore, reallocating 10 min from sleep to SB resulted in a significant decrease in BMI z-scores (−0.04; 95% CI [−0.06, −0.01]), while adding 10 min of sleep by reducing SB significantly increased BMI z-scores (0.04; 95% CI [0.01, 0.06]).

**Table 3:** Cross-sectional associations between the composition of 24-hour movement behaviors and social-emotional development, gross motor development, and BMI z-scores

Outcome	*n*	Composition				ilr1-PA		ilr1-SB		ilr1-Sleep	
		F (Res.df, df)	*p*	*R*²Δ	*R*²	β (95% CI)	*p*	β (95% CI)	*p*	β (95% CI)	*p*
SE raw score	105	0.94 (100, 2)	.393	.001	.922	1.19 (−4.79, 7.18)	.693	7.81 (−3.56. 19.19)	.176	−9.00 (−23.84, 5.83)	.231
SE scaled score	104	1.32 (99, 2)	.273	.024	.083	−0.49 (−1.86, 0.89)	.483	1.44 (−1.18, 4.06)	.278	−0.95 (−4.33, 2.43)	.577
GM raw score	62	**10.06 (56**,** 2)**	**< .001**	.042	.880	0.93 (−1.92, 3.77)	.517	**−9.43 (**−**15.11**, −**3.75)**	**.002**	**8.51 (0.90**,** 16.11)**	.029*
GM scaled score	61	0.01 (58, 2)	.986	.000	.056	0.16 (−1.92, 2.25)	.876	0.05 (−3.70, 3.80)	.979	−0.21 (−5.39, 4.96)	.935
BMI z-score	63	**4.55 (58**,** 2)**	**.015**	.130	.171	−0.18 (−0.62, 0.27)	.430	**−1.28 (−2.16**,** −3.40)**	**.005**	**1.46 (0.29**,** 2.63)**	**.016**

Abbreviations: BMI body mass index, CI confidence interval, GM gross motor development, ilr isometric log ratio, PA physical activity, SB sedentary behavior, SE social-emotional development

**Bold fonts** indicate *p* < .05

Note: The child’s age and sex were included as covariates in all linear regression models.

*Not significant before removal of influential measurements based on Cook’s distance and studentized residual values

**Table 4:** Longitudinal associations between the compositions of 24-hour movement behaviors and social-emotional development, gross motor development, and BMI z-scores

Outcome	*n*	Composition				ilr1-PA		ilr1-SB		ilr1-Sleep	
		X^2^	*p*	*R*²Δ	*R*² mar/con	β (95% CI)	*p*	β (95% CI)	*p*	β (95% CI)	*p*
SE raw score	107	3.27	.195	.0004	.885/.945	1.41 (−3.60, 6.43)	.580	9.77 (−0.93, 20.47)	.077*	−11.18 (−24.93, 2.57)	.114*
SE scaled score	104	2.69	.261	.017	.068/.251	0.13 (−1.03, 1.29)	.828	1.82 (−0.48, 4.12)	.125	−1.94 (−4.90, 1.02)	.200
GM raw score	62	**19.88**	**< .001**	.033	.848/.855	−1.79 (−4.19, 0.60)	.148	**−11.27 (−16.11**,** −6.42)**	**< .001**	**13.06 (6.67**,** 19.50)**	**< .001**
GM scaled score	61	2.92	.233	.029	.094/.341	−1.23 (−2.77, 0.28)	.115	−1.79 (−4.70, 1.12)	.232	3.01 (−0.86, 6.89)	.133
BMI z-score	63	5.63	.060	.050	.093/.524	−0.29 (−0.67, 0.09)	.136	**−0.91 (−1.69**,** −0.13)**	**.027**	**1.20 (0.18**,** 2.23)**	**.026**

Abbreviations: BMI body mass index, CI confidence interval, con conditional, GM gross motor development, ilr isometric log ratio, mar marginal, PA physical activity, SB sedentary behavior, SE social-emotional development

**Bold fonts** indicate *p* < .05

Note: The child’s age and sex at both timepoints were included as fixed effects in all linear mixed models.

*Significant before removal of influential measurements based on Cook’s distance and studentized residual values

**Table 5:** Predicted changes in Raw gross motor development scores and BMI z-scores after compositional isotemporal reallocations of 10 min between movement behaviors, using one-by-one reallocations

Outcome	PA to SB	PA to sleep	SB to PA	SB to sleep	Sleep to PA	Sleep to SB
GM raw score (95% CI)	−**0.22 (**−**0.33**, −**0.11)**	0.04 (−0.15, 0.23)	**0.22 (0.11**,** 0.33)**	**0.27 (0.08**,** 0.45)**	−0.05 (−0.23, 0.14)	−**0.26 (**−**0.45**, −**0.08)**
BMI z-score (95% CI)	−0.02 (−0.03, 0.00)	0.02 (−0.01, 0.05)	0.02 (0.00, 0.03)	**0.04 (0.01**,** 0.06)**	−0.02 (−0.05, 0.01)	−**0.04 (**−**0.06**,** −0.01)**

Abbreviations: BMI body mass index, CI confidence interval, GM gross motor development, PA physical activity, SB, sedentary behavior

**Bold fonts** indicate *p* < .05

## Discussion

This study examined both cross-sectional and longitudinal associations of the composition of 24-hour movement behaviors with growth, social-emotional development, and gross motor development in children aged 0–4 years. We observed significant associations of the composition of 24-hour movement behaviors with raw motor development scores, both cross-sectionally and longitudinally, and with BMI z-scores, cross-sectionally. No significant associations were observed between 24-hour movement behavior composition and social-emotional development. Reallocating time from SB to PA or sleep significantly improved predicted raw motor development scores, while reallocating time from sleep to SB significantly improved BMI z-scores.

The proportions of time spent in PA, SB, and sleep in our sample ranged from 13.2–15.7%, 28.4–32.2%, and 54.5–55.9%, respectively. Our findings differ from those of Taylor et al. (2018), who reported that children aged 1–5 years spent less than 50% of their day asleep and more than 20% in PA [47]. These differences could potentially be explained by the slightly older sample and the use of accelerometers by Taylor et al. versus parent-report in our study. At the second timepoint the proportions in our sample changed slightly, with SB increasing by approximately 2% at the expense of PA and sleep. This shift may be attributed to the children who were not followed up at the second timepoint because they had turned four years old. On average, these older children engaged in more PA and less sleep than the younger ones, which likely influenced the overall distribution of behaviors in our sample.

We found no significant association between the composition of 24-hour movement behaviors and the scaled gross motor development scores. However, as hypothesized, higher relative SB was associated with poorer raw gross motor development scores, while higher relative sleep was associated with better gross motor development scores. Specifically, in compositional substitutional reallocation models, we found that replacing 10 min of SB by PA or sleep increased raw gross motor development scores by 0.22 or 0.27, respectively. Kuzik et al. (2020) and Mota et al. (2020) previously investigated cross-sectional associations between accelerometer-based 24-hour movement behaviors and motor development in preschoolers, specifically focusing on locomotor skills, object control skills, and total motor skills [46, 48]. Both studies found a positive association of moderate-to-vigorous PA (MVPA) with motor development, relative to other movement behaviors within the composition [46, 48]. Similarly, Song et al. (2024) found a prospective positive association between MVPA, relative to the other behaviors, and locomotor and ball skills, while light-intensity PA (LPA) was negatively associated with locomotor skills [49]. Additionally, Kuzik et al. (2020) and Mota et al. (2020) found that increasing SB by decreasing MPVA resulted in poorer motor developmental outcomes [46, 48]. However, both studies also reported that increasing SB by decreasing LPA improved motor skills. Furthermore, contrary to our findings, Mota et al. found a decrease in motor skills when replacing SB by sleep, while Kuzik et al. found no significant changes [46, 48].

Comparison between these studies and our study is challenging due to several reasons. First, our sample was aged 0–4 years, whereas earlier research focused on 3–5 year-olds [46, 48, 49]. Second, our study is based on parent-report while aforementioned studies used accelerometer data. Third, we did not differentiate between LPA and MVPA as classifying PA intensities is very challenging for infants and toddlers due to the lack of established metabolic equivalents, and clear PA definitions [30, 50, 51]. Future research should address these challenges in accurate assessment of 24-hour movement behaviors in young children. Despite these differences, the lack of a consistent relationship between 24-hour movement behavior composition and gross motor development remains across studies. Therefore, larger longitudinal studies are essential to further investigate how movement behavior compositions are associated with motor development in infants, toddlers, and preschoolers.

Because norm-referenced scores for social-emotional and motor development were unavailable for children older than 42 months and 15 days, we were unable to compute scaled scores for 15 children [33]. Therefore, we also conducted analyses with total raw scores including the total sample of children, adjusting for age and sex. Consequently, the results from the raw scores should be interpreted cautiously. However, by including age and sex as covariates in our analysis, we aimed to reduce potential bias. The observed association with raw motor development scores, but not with scaled scores, may be attributed to the greater variability in the raw scores, which could increase sensitivity to differences between children.

The mean BMI z-scores in our sample (0.7–1.0) were relatively high. A score of +1 is classified as at risk for overweight, while a score of +2 indicates overweight [52]. These elevated scores may largely be attributed to the light clothing worn by children during weight measurements. Since no standardized corrections exist for adjusting weight based on clothing in children aged 0–4 years, we did not apply any adjustments [52, 53]. Future studies are recommended to, whenever possible, remove outer clothing during weight measurements to minimize potential bias.

We found a significant association between movement behavior composition and BMI z-scores in the cross-sectional analysis only. Contrary to our hypothesis, we found that higher relative sleep was associated with higher BMI z-scores. Additionally, unexpectedly, we found that higher relative SB, was associated with lower BMI z-scores. Specifically, we found that reallocating 10 min from sleep to SB resulted in a decrease in predicted BMI z-scores of −0.04. We found no significant association between relative PA and BMI z-scores. Previously, Taylor et al. (2018) found a cross-sectional association between the composition of 24-hour movement behaviors and BMI z-scores for 3.5-year-old children, but not for those aged 1, 2, or 5 years [54]. Additionally, no longitudinal associations were found [54]. In line with our findings, Carson et al. (2017) and Decraene et al. (2025) found a significant association between the composition of movement behaviors and BMI z-scores in preschoolers, while Kuzik et al. (2020) and McGee et al. (2020) found no association [45, 48, 55, 56]. Additionally, findings on the relative contribution of specific movement behaviors within the composition on the association with BMI z-scores remains inconsistent across studies. One study found no association [56], while others reported that higher relative sleep [45, 54] was associated with lower BMI z-scores, while higher relative SB [54], LPA [54], and MVPA [45] were associated with higher BMI z-scores. Additionally, Kuzik et al. (2020) found that reallocating time from SB to MVPA led to an increase in predicted BMI z-scores [48]. Similarly, Decraene et al. (2025) reported comparable results when reallocating either SB or sleep to MVPA [45]. The increase in BMI z-scores with higher PA found in previous studies, as well as the decrease in BMI z-scores with higher SB in our study, may seem counterintuitive. Decraene et al. (2025) suggested that increased PA may contribute to fat-free mass in preschoolers, which could explain the unexpected increase in BMI z-scores [45, 57]. However, the changes in BMI z-scores observed in both our study and previous research appear small and may not be clinically relevant. Thus, evidence on the optimal distribution of time spent in each movement behavior in young children remains inconclusive. Potentially, the adverse effects of unhealthy movement behaviors may only develop over longer time periods [58]. Furthermore, as mostly significant cross-sectional associations have been reported, the causal direction of the relationship between 24-hour movement behaviors and BMI remains unclear. Therefore, longitudinal studies over extended periods are essential to advance research in this field. Additionally, to better understand the relationship between movement behavior composition and healthy growth in young children, future studies should incorporate additional indicators of body composition (e.g., skinfold thickness).

The association between the composition of 24-hour movement behaviors and social-emotional development in early childhood has previously been studied by Kuzik et al. (2020) and St. Laurent et al. (2023) [48, 59]. Despite methodological differences with our study (e.g., study design, age range, measurement instruments), these compositional analysis studies found no significant associations with social-emotional development either [48, 59]. Future studies are recommended to further explore the social context and types of activities relevant to children’s social-emotional development. For example, within SB, activities such as being read to by an adult may support social-emotional development, whereas unmonitored screen time may have an adverse impact [23, 60].

### Strengths and limitations

This study has several strengths and limitations. Strengths include the longitudinal data and the compositional analysis of 24-hour movement behaviors as well as data in the full 0–4 age range. The use of the Bayley-III-NL to assess social-emotional and gross motor development is another strength of our study, as this is a widely validated instrument specifically designed for this age group [32, 34].

A limitation is the considerable dropout. In sub-cohort-SE, complete data were available for 52.3% of the children at the first timepoint and 39.5% at the second. Similarly, in sub-cohort-GM, complete data were available for 72.9% of the children at the first timepoint, and 65.3% at the second. The primary cause of missing data was non-response despite initial consent, potentially due to the perceived burden of using the My Little Moves app. This reduced sample size limits the study’s statistical power. Consequently, we were also unable to differentiate between age subgroups, limiting our ability to explore potential variations in associations across developmental phases. Another limitation is the reliance on a parent-reported time-use diary to assess children’s 24-hour movement behaviors, which may be affected by recall bias and social desirability bias. As many young children in the Netherlands attend daycare centers [61], parents are unable to report activities performed during these periods. Furthermore, the mean interval between the first and second timepoint of approximately nine months, as well as the relatively small difference in mean age of children between the two timepoints (5.1 months for sub-cohort SE and 7.2 months for sub-cohort GM) may limit the ability to capture developmental changes. Also, although mean age and sex were similar between the two sub-cohorts, collecting data from two separate samples limits our ability to directly compare outcomes due to potential differences between the groups. Additionally, the majority of participating parents were highly educated, limiting the generalizability of our findings. Moreover, conducting compositional isotemporal reallocation analyses only to the cross-sectional data is a limitation. Nevertheless, the significant associations found for the irl1 coordinates were largely consistent across both the cross-sectional and longitudinal analyses, in terms of both direction and magnitude. Therefore, we expect that the findings would have been comparable. Future studies are recommended to further develop and apply this type of analysis in longitudinal studies. Lastly, due to limited statistical power, only the child’s age and sex were included as covariates in the analyses. Thus our models did not adjust for other potential confounding effects such as children’s ethnicity, parental education, or income [48].

## Conclusions

Our findings indicate significant associations between the composition of 24-hour movement behaviors and both BMI and gross motor development in children aged 0–4 years, but not with social-emotional development. Specifically, more SB, relative to other movement behaviors, was associated with lower BMI z-scores and raw gross motor development scores, while more sleep, relative to other behaviors, was associated with higher BMI z-scores and raw motor development scores. However, PA, relative to other behaviors, was not significantly associated with neither BMI z-scores nor development scores.

## Supplementary Information

Below is the link to the electronic supplementary material.

Supplementary Material 1

Supplementary Material 2

## Data Availability

The R scripts used for analyses of data from this study are available in Additional file 2. Data that support the findings of the study are available from the corresponding author upon reasonable request.

## Abbreviations

App: application
Bayley-III-NL: Bayley Scales of Infant and Toddler Development-Third Edition
BMI: body mass index
CI: confidence interval
ECEC: early childhood education and care
GM: gross motor development
Ilr: isometric log-ratio
LPA: light physical activity
MVPA: moderate-to-vigorous physical activity
PA: physical activity
SB: sedentary behavior
SE: social-emotional development
sub-cohort-GM: sub-cohort examining growth and gross motor development
sub-cohort-SE: sub-cohort examining social-emotional development
WHO: World Health Organization
zBMI: BMI z-score

## References

[1] Likhar A, Baghel P, Patil M. Early childhood development and social determinants. Cureus. 2022;14(9):e29500. 36312682 10.7759/cureus.29500 PMC9596089

[2] Council NR. From neurons to neighborhoods: The science of early childhood development. 2000. 25077268

[3] Cusick SE, Georgieff MK. The role of nutrition in brain development: the golden opportunity of the first 1000 days. J Pediatr. 2016;175:16–21. 27266965 10.1016/j.jpeds.2016.05.013 PMC4981537

[4] Rafiyya A, Kraiwanit T, Limna P, Sonsuphap R, Kasrisom A, Snongtaweeporn T. Early childhood social-emotional development: an impact on a developing country. Int J Evaluation Res Educ. 2024;13(5):3081–9.

[5] Katagiri M, Ito H, Murayama Y, Hamada M, Nakajima S, Takayanagi N, et al. Fine and gross motor skills predict later psychosocial maladaptation and academic achievement. Brain Develop. 2021;43(5):605–15. 10.1016/j.braindev.2021.01.003 33558106

[6] Utesch T, Bardid F, Büsch D, Strauss B. The relationship between motor competence and physical fitness from early childhood to early adulthood: a meta-analysis. Sports Med. 2019;49:541–51. 30747376 10.1007/s40279-019-01068-y

[7] n der Fels IMJ, te Wierike SCM, Hartman E, Elferink-Gemser MT, Smith J, Visscher C. The relationship between motor skills and cognitive skills in 4–16 year old typically developing children: A systematic review. J Sci Med Sport. 2015;18(6):697–703. 25311901 10.1016/j.jsams.2014.09.007

[8] Piek JP, Dawson L, Smith LM, Gasson N. The role of early fine and gross motor development on later motor and cognitive ability. Hum Mov Sci. 2008;27(5):668–81. 18242747 10.1016/j.humov.2007.11.002

[9] Maffeis C, Tatò L. Long-term effects of childhood obesity on morbidity and mortality. Horm Res. 2001;55(Suppl 1):42–5. 11408761 10.1159/000063462

[10] Reilly JJ, Kelly J. Long-term impact of overweight and obesity in childhood and adolescence on morbidity and premature mortality in adulthood: systematic review. Int J Obes. 2011;35(7):891–8. 10.1038/ijo.2010.222 20975725

[11] Carson V, Lee E-Y, Hewitt L, Jennings C, Hunter S, Kuzik N, et al. Systematic review of the relationships between physical activity and health indicators in the early years (0–4 years). BMC Public Health. 2017;17(5):33–63. 29287590 10.1186/s12889-017-4981-5 PMC5747177

[12] Rollo S, Antsygina O, Tremblay MS. The whole day matters: Understanding 24-hour movement guideline adherence and relationships with health indicators across the lifespan. J Sport Health Sci. 2020;9(6):493–510. 32711156 10.1016/j.jshs.2020.07.004 PMC7749249

[13] Tremblay MS. Introducing 24-hour movement guidelines for the early years: a new paradigm gaining momentum. J Phys Activity Health. 2020;17(1):92–5. 10.1123/jpah.2019-0401 31711035

[14] Organization WH. Guidelines on physical activity, sedentary behaviour and sleep for children under 5 years of age. World Health Organization; 2019. 31091057

[15] Willumsen J, Bull F. Development of WHO guidelines on physical activity, sedentary behavior, and sleep for children less than 5 years of age. J Phys Activity Health. 2020;17(1):96–100. 10.1123/jpah.2019-0457 31877559

[16] Bianconi A, Fiore M, Zauli E, Acuti Martellucci C, Rosso A, Dallolio L et al. How strong is the evidence supporting the WHO guidelines on physical activity, sedentary behaviour and sleep in early childhood? Eur J Clin Invest. 2024:e14294. 10.1111/eci.14294 39086022

[17] Veldman SL, Chin A, Paw MJ, Altenburg TM. Physical activity and prospective associations with indicators of health and development in children aged < 5 years: a systematic review. Int J Behav Nutr Phys Activity. 2021;18:1–11. 10.1186/s12966-020-01072-w PMC7791660 33413484

[18] Poitras VJ, Gray CE, Janssen X, Aubert S, Carson V, Faulkner G, et al. Systematic review of the relationships between sedentary behaviour and health indicators in the early years (0–4 years). BMC Public Health. 2017;17:65–89. 29219092 10.1186/s12889-017-4849-8 PMC5773886

[19] Chaput J-P, Gray CE, Poitras VJ, Carson V, Gruber R, Birken CS, et al. Systematic review of the relationships between sleep duration and health indicators in the early years (0–4 years). BMC Public Health. 2017;17:91–107. 29219078 10.1186/s12889-017-4850-2 PMC5773910

[20] Kuzik N, Poitras VJ, Tremblay MS, Lee E-Y, Hunter S, Carson V. Systematic review of the relationships between combinations of movement behaviours and health indicators in the early years (0–4 years). BMC Public Health. 2017;17(5):109–22. 29219071 10.1186/s12889-017-4851-1 PMC5773877

[21] Zahran S, Visser C, Ross-White A, Janssen I. A systematic review of compositional analysis studies examining the associations between sleep, sedentary behaviour, and physical activity with health indicators in early childhood. J Activity Sedentary Sleep Behav. 2023;2(1):1–11. 10.1186/s44167-022-00012-2 PMC11960365 40217382

[22] Carson V, Draper CE, Okely A, Reilly JJ, Tremblay MS. Future directions for movement behavior research in the early years. J Phys Activity Health. 2023;1(aop):1–4. 10.1123/jpah.2023-0679 38109880

[23] Carson V, Zhang Z, Predy M, Pritchard L, Hesketh KD. Longitudinal associations between infant movement behaviours and development. Int J Behav Nutr Phys Activity. 2022;19(1):1–15. 10.1186/s12966-022-01248-6 PMC8800227 35090492

[24] Chastin SF, Palarea-Albaladejo J, Dontje ML, Skelton DA. Combined effects of time spent in physical activity, sedentary behaviors and sleep on obesity and Cardio-Metabolic health markers: A novel compositional data analysis approach. PLoS ONE. 2015;10(10):e0139984. 26461112 10.1371/journal.pone.0139984 PMC4604082

[25] Dumuid D, Pedišić Ž, Palarea-Albaladejo J, Martín-Fernández JA, Hron K, Olds T. Compositional data analysis in Time-Use epidemiology: what, why, how. Int J Environ Res Public Health. 2020;17(7). 10.3390/ijerph17072220 PMC7177981 32224966

[26] Altenburg TM, Gubbels JS, Arts J, Lettink A, Veldman S, Verhoeff A, et al. 24-hour movement behaviours in the early years, potential behavioural determinants and prospective associations with growth, motor and social–emotional development: the my little moves study protocol. BMJ Open. 2024;14(10):e081836. 39438091 10.1136/bmjopen-2023-081836 PMC11499838

[27] von Elm E, Altman DG, Egger M, Pocock SJ, Gøtzsche PC, Vandenbroucke JP. The strengthening the reporting of observational studies in epidemiology (STROBE) statement: guidelines for reporting observational studies. Lancet. 2007;370(9596):1453–7. 18064739 10.1016/S0140-6736(07)61602-X

[28] Ujcic-Voortman J, Hall J, Johannes M, Seidell J, Verhoeff A. Sarphati amsterdam: a dynamic research infrastructure. Eur J Pub Health. 2020;30(Supplement5):ckaa165.

[29] Statistics UIf. International standard classification of education: ISCED 2011. Comp Social Res. 2012;30.

[30] Arts J, Chinapaw MJM, Gubbels JS, Verhoeff AP, Brons A, Veldman S, et al. Development and content validity of an application to assess 24-hour movement behaviors in 0–4-year-old children involving end-users and key stakeholders: the my little moves app. Int J Behav Nutr Phys Activity. 2024;21(1):2. 10.1186/s12966-023-01552-9 PMC10763169 38167442

[31] Lettink A, Arts J, Gubbels JS, Altenburg TM, Chinapaw MJM. Assessing 24-h movement behaviors in early childhood (0–4 years): reliability of the my little moves app and comparison with accelerometry. J Activity Sedentary Sleep Behav. 2025;4(1):5. 10.1186/s44167-025-00085-9 PMC12392550 40877997

[32] Steenis LJ, Verhoeven M, Hessen DJ, van Baar AL. First steps in developing the Dutch version of the Bayley III: is the original Bayley III and its item sequence also adequate for Dutch children? Eur J Dev Psychol. 2014;11(4):494–511.

[33] Van Baar AS, Verhoeven LJP, Hessen M, Smits-Engelsman DJ. BCM. Bayley-III-NL, technische handleiding. Amsterdam: Pearson Benelux B.V.; 2015.

[34] Griffiths A, Toovey R, Morgan PE, Spittle AJ. Psychometric properties of gross motor assessment tools for children: a systematic review. BMJ Open. 2018;8(10):e021734. 30368446 10.1136/bmjopen-2018-021734 PMC6224743

[35] Crespi CM, Alfonso VH, Whaley SE, Wang MC. Validity of child anthropometric measurements in the special supplemental nutrition program for women, infants, and children. Pediatr Res. 2012;71(3):286–92. 22337260 10.1038/pr.2011.37 PMC3282987

[36] Schumacher D, Borghi E, Polonsky J, Schumacher MD. Package ‘anthro’. Computation of the WHO child growth standards. CRAN; 2023. https://github.com/dirkschumacher/anthro.

[37] n den Boogaart KG, Tolosana R, van den Bren M. Package ‘compositions’. Compositional Data Anal Ver. 2013;1:40–1.

[38] Belsley DA, Kuh E, Welsch RE. Regression diagnostics: identifying influential data and sources of collinearity. Wiley; 2005.

[39] Pinheiro J, Bates D, DebRoy S, Sarkar D, Heisterkamp S, Van Willigen B, et al. Package ‘nlme’. Linear and nonlinear mixed effects models. Version. 2017;3(1):274.

[40] Gelman A. Data analysis using regression and multilevel/hierarchical models. Cambridge University Press; 2007.

[41] Twisk J, de Boer M, de Vente W, Heymans M. Multiple imputation of missing values was not necessary before performing a longitudinal mixed-model analysis. J Clin Epidemiol. 2013;66(9):1022–8. 23790725 10.1016/j.jclinepi.2013.03.017

[42] Cohen J. Statistical power analysis for the behavioral sciences. Routledge; 2013.

[43] Dumuid D, Pedišić Ž, Stanford TE, Martín-Fernández JA, Hron K, Maher CA, et al. The compositional isotemporal substitution model: A method for estimating changes in a health outcome for reallocation of time between sleep, physical activity and sedentary behaviour. Stat Methods Med Res. 2019;28(3):846–57. 29157152 10.1177/0962280217737805

[44] Stanford T, Rasmussen CL, Dumuid D. codaredistlm: compositional data linear models with composition redistribution_. R package version 0.1. 0. 2022.

[45] Decraene M, Chong KH, Stanford T, Dumuid D, Cross P, Cardon G, et al. The association between 24-h movement behaviours and adiposity among Australian preschoolers: a compositional data analysis. BMC Public Health. 2025;25(1):368. 39881264 10.1186/s12889-024-21217-x PMC11781000

[46] Mota JG, Clark CCT, Bezerra TA, Lemos L, Reuter CP, Mota JAPS, et al. Twenty-four-hour movement behaviours and fundamental movement skills in preschool children: A compositional and isotemporal substitution analysis. J Sports Sci. 2020;38(18):2071–9. 32508219 10.1080/02640414.2020.1770415

[47] Liu T, Benjamin-Neelon SE. A longitudinal study of infant 24-hour sleep: comparisons of sleep diary and accelerometer with different algorithms. Sleep. 2023;46(11). 10.1093/sleep/zsad160 PMC10639156 37279933

[48] Kuzik N, Naylor P-J, Spence JC, Carson V. Movement behaviours and physical, cognitive, and social-emotional development in preschool-aged children: Cross-sectional associations using compositional analyses. PLoS ONE. 2020;15(8):e0237945. 32810172 10.1371/journal.pone.0237945 PMC7433874

[49] Song H, Lau PWC, Wang JJ, Zhou P, Shi L. Prospective association between 24-Hour movement behaviors and fundamental movement skills in Chinese preschoolers during the COVID-19 pandemic: A compositional and reallocation analysis. J Phys Act Health. 2024;21(11):1167–73. 39348880 10.1123/jpah.2024-0142

[50] Butte NF, Watson KB, Ridley K, Zakeri IF, McMurray RG, Pfeiffer KA, et al. A youth compendium of physical activities: activity codes and metabolic intensities. Med Sci Sports Exerc. 2018;50(2):246. 28938248 10.1249/MSS.0000000000001430 PMC5768467

[51] Truelove S, Vanderloo LM, Tucker P. Defining and measuring active play among young children: a systematic review. J Phys Activity Health. 2017;14(2):155–66. 10.1123/jpah.2016-0195 27775475

[52] Organization WH. Training course on child growth assessment. Geneva: WHO. 2008:17–25.

[53] Hollanders J. Gewicht Van Kleding Van kinderen Bij Het periodiek geneeskundig Onderzoek Groep 2. JGZ Tijdschrift Voor Jeugdgezondheidszorg. 2022;54(5):98–100.

[54] Taylor RW, Haszard JJ, Meredith-Jones KA, Galland BC, Heath A-LM, Lawrence J, et al. 24-h movement behaviors from infancy to preschool: cross-sectional and longitudinal relationships with body composition and bone health. Int J Behav Nutr Phys Activity. 2018;15(1):118. 10.1186/s12966-018-0753-6 PMC6260686 30477518

[55] McGee M, Unger S, Hamilton J, Birken CS, Pausova Z, Vanderloo LM, et al. Lean mass accretion in children born very low birth weight is significantly associated with estimated changes from sedentary time to light physical activity. Pediatr Obes. 2020;15(5):e12610. 31914236 10.1111/ijpo.12610

[56] Carson V, Tremblay MS, Chastin SFM. Cross-sectional associations between sleep duration, sedentary time, physical activity, and adiposity indicators among Canadian preschool-aged children using compositional analyses. BMC Public Health. 2017;17(5):848. 29219077 10.1186/s12889-017-4852-0 PMC5773862

[57] Butte NF, Puyau MR, Wilson TA, Liu Y, Wong WW, Adolph AL, et al. Role of physical activity and sleep duration in growth and body composition of preschool-aged children. Obesity. 2016;24(6):1328–35. 27087679 10.1002/oby.21489 PMC4882246

[58] Proctor MH, Moore LL, Gao D, Cupples LA, Bradlee ML, Hood MY, et al. Television viewing and change in body fat from preschool to early adolescence: the Framingham children’s study. Int J Obes. 2003;27(7):827–33. 10.1038/sj.ijo.0802294 12821969

[59] St. Laurent CW, Rasmussen CL, Holmes JF, Cremone-Caira A, Kurdziel LBF, Desrochers PC, et al. Associations of activity, sedentary, and sleep behaviors with cognitive and social-emotional health in early childhood. J Activity Sedentary Sleep Behav. 2023;2(1):7. 10.1186/s44167-023-00016-6 PMC11116218 38798902

[60] Zhao J, Yu Z, Sun X, Wu S, Zhang J, Zhang D, et al. Association between screen time trajectory and early childhood development in children in China. JAMA Pediatr. 2022;176(8):768–75. 35666518 10.1001/jamapediatrics.2022.1630 PMC9171655

[61] HOVIUS L. Kosten En Gebruik formele kinderopvang Zijn afgelopen Jaren Toegenomen. KINDEROPVANG.30.

