# Supplementary material for: Cross-sectional and longitudinal associations between 24-hour movement behaviors and growth, motor, and social-emotional development in early childhood
Source: J Act Sedentary Sleep Behav. 2025 Aug 28;4:14. doi: 10.1186/s44167-025-00085-9 (PMC12392550; doi:10.1186/s44167-025-00085-9)
Supplement: Supplementary file 2 — Supplementary Material 2 [file 44167_2025_85_MOESM2_ESM.pdf]

Additional file 2

Cross-sectional and longitudinal associations between 24-hour movement behaviors and growth, motor, and social-emotional development in early childhood

Jelle Arts [1,2], Teatske M. Altenburg [1,2,3], Annelinde Lettink [1,2,3], Arnoud P. Verhoeff [,4, 5], Jessica S. Gubbels [6], Mai J. M. Chinapaw [1,2,3]

[1] Amsterdam UMC location Vrije Universiteit Amsterdam, Public and Occupational Health, De Boelelaan 1117, Amsterdam, The Netherlands;

[2] Amsterdam Public Health, Health Behaviors & Chronic Diseases, Amsterdam, The Netherlands;

[3] Amsterdam Public Health, Methodology, Amsterdam, The Netherlands;

[4] Public Health Service Amsterdam, Sarphati Amsterdam, 1018 WT Amsterdam, The Netherlands;

[5] Department of Sociology, University of Amsterdam, 1018 WV Amsterdam, The Netherlands;

[6] Maastricht University, Department of Health Promotion, NUTRIM Institute of Nutrition and Translational Research in Metabolism, PO Box 616, 6200 MD Maastricht, The Netherlands.

Corresponding author:

Jelle Arts

j.arts@amsterdamumc.nl

R-Script for:

1. Calculating ILR-coordinates;
2. Longitudinal compositional data analysis: Linear mixed model;
3. Cross-sectional compositional data analysis: linear regression;
4. Compositional isotemporal reallocation analysis.

##### 1 Calculating ILR-coordinates

```
install.packages("compositions") # For working with compositional data
library(compositions)

# Calculate total time (sum of PA, SB, and Sleep)
data$total_time <- data$PA_mean + data$SB_mean + data$Sleep_mean

# Calculate the proportions
data$PA_prop <- data$PA_mean / data$total_time
data$SB_prop <- data$SB_mean / data$total_time
data$Sleep_prop <- data$Sleep_mean / data$total_time

# Calculate ILR-coordinates using the compositions package
proportions.sleep <- acomp(cbind(data$PA_prop, data$SB_prop, data$Sleep_prop))
colnames(proportions.sleep) <- c("PA_prop", "SB_prop", "Sleep_prop")
ilr.sleep <- ilr(proportions.sleep)
colnames(ilr.sleep) <- c("ilr2.sleep", "ilr1.sleep")
ilr.sleep <- as.data.frame(ilr.sleep)
data$ILR1_Sleep <- ilr.sleep$ilr1.sleep
data$ILR2_Sleep <- ilr.sleep$ilr2.sleep

proportions.sb <- acomp(cbind(data$PA_prop, data$Sleep_prop, data$SB_prop))
colnames(proportions.sb) <- c("PA_prop", "Sleep_prop", "SB_prop")
ilr.sb <- ilr(proportions.sb)
colnames(ilr.sb) <- c("ilr2.sb", "ilr1.sb")
ilr.sb <- as.data.frame(ilr.sb)
data$ILR1_SB <- ilr.sb$ilr1.sb
data$ILR2_SB <- ilr.sb$ilr2.sb

proportions.pa <- acomp(cbind(data$SB_prop, data$Sleep_prop, data$PA_prop))
colnames(proportions.pa) <- c("SB_prop", "Sleep_prop", "PA_prop")
ilr.pa <- ilr(proportions.pa)
colnames(ilr.pa) <- c("ilr2.pa", "ilr1.pa")
ilr.pa <- as.data.frame(ilr.pa)
data$ILR1_PA <- ilr.pa$ilr1.pa
data$ILR2_PA <- ilr.pa$ilr2.pa

# Define ILR sets
ILR_sets <- list(
  c("ILR1_PA", "ILR2_PA"),
  c("ILR1_SB", "ILR2_SB"),
  c("ILR1_Sleep", "ILR2_Sleep")
)
```

##### 2 Longitudinal compositional data analysis: Linear mixed model

```
#install.packages("nlme") # For mixed models
#install.packages("MuMIn") # For R^2 computation in mixed models

library(nlme)
library(MuMIn)

# Function to run the mixed model for specific score and ILR variables
run_mixed_model <- function(score_type, ILR_vars) {

  # 1. Build the null model without ILR variables
  formula_null <- as.formula(paste(score_type, "~ gender_child + age_child_months"))
  model_null <- lme(formula_null,
                random = ~ 1 | castorID,
                data = data,
                method = "ML",
                na.action = na.exclude)


  # Calculate marginal R^2 for the null model
  R2_baseline <- r.squaredGLMM(model_null)[1]  # Marginal R^2
  R2_conditional_baseline <- r.squaredGLMM(model_null)[2]  # Conditional R^2

  # 2. Build the full model with ILR variables
  formula_full <- as.formula(paste(score_type, "~", paste(ILR_vars, collapse = " + "), "+ gender_child + age_child_months"))
  model_full <- lme(formula_full,
                random = ~ 1 | castorID,
                data = data,
                method = "ML",
                na.action = na.exclude)

  assign("model_full", model_full, envir = .GlobalEnv)

  # Calculate marginal R^2 for the full model
  R2_full <- r.squaredGLMM(model_full)[1]  # Marginal R^2
  R2_conditional_full <- r.squaredGLMM(model_full)[2]  # Conditional R^2

  # 3. Calculate R^2 change
  R2_change <- R2_full - R2_baseline

  # Results from the model: fixed effects and confidence intervals
  fixed_effects <- summary(model_full)$tTable
  ci_fixed_effects <- intervals(model_full, level = 0.95)$fixed

  # Display the model summary
  cat("\nModel summary for", score_type, "with ILR variables", paste(ILR_vars, collapse = ", "), ":\n")
  print(summary(model_full))

  # Calculate and display the 95% confidence interval for the fixed effects
  cat("\n95% confidence interval for fixed effects:\n")
  print(ci_fixed_effects)

  # Run the null model (without ILR variables)
  formula_null <- as.formula(paste(score_type, "~ gender_child + age_child_months"))
  model_null <- lme(formula_null,
```

```
                    random = ~ 1 | castorID,
                    data = data,
                    method = "ML",
                    na.action = na.exclude)


  # Compare models with an ANOVA test to test the effect of the ILR coordinate set
  cat("\nANOVA result for", score_type, "with ILR variables", paste(ILR_vars, collapse = ", "), ":\n")
  anova_result <- stats::anova(model_full, model_null)
  print(anova_result)
}

# Run the analysis for the different score types and ILR sets
for (ILR_vars in ILR_sets) {
  run_mixed_model("raw_score_SE", ILR_vars)
  run_mixed_model("scaled_score_SE", ILR_vars)
  run_mixed_model("raw_score_GMS", ILR_vars)
  run_mixed_model("sclaled_score_GMS", ILR_vars)
  run_mixed_model("zbmi", ILR_vars)
}
```

##### 3 Cross-sectional compositional data analysis: linear regression

```
# Function to get the first measurement per participant
get_first_measurement <- function(data) {
  sorted_data <- data[order(data$castorID, data$measurement), ]
  first_measurement <- sorted_data[!duplicated(sorted_data$castorID), ]
  return(first_measurement)
}

# Get filtered data with only the first measurement
first_measurement <- get_first_measurement(data)

# Function for linear regression analysis
run_regression <- function(score_type, ILR_vars, data) {
  complete_data <- data[!is.na(data[[ILR_vars[1]]]) & !is.na(data[[ILR_vars[2]]]) & !is.na(data[[score_type]]), ]

  formula_full <- as.formula(paste(score_type, "~", paste(ILR_vars, collapse = " + "), "+ gender_child + age_child_months"))
  model_full <- lm(formula_full, data = complete_data, na.action = na.exclude)

  formula_null <- as.formula(paste(score_type, "~ gender_child + age_child_months"))
  model_null <- lm(formula_null, data = complete_data, na.action = na.exclude)

  R2_change <- summary(model_full)$r.squared - summary(model_null)$r.squared
  anova_result <- anova(model_null, model_full)

  cat("\nANOVA result for", score_type, "with ILR variables", paste(ILR_vars, collapse = ", "), ":\n")
  print(anova_result)

  # Print full model summary
  cat("\nFull model summary for", score_type, "with ILR variables", paste(ILR_vars, collapse = ", "), ":\n")
  print(summary(model_full))
}

# Run regression for different score types and ILR sets
for (ILR_vars in ILR_sets) {
  run_regression("raw_score_SE", ILR_vars, first_measurement)
  run_regression("scaled_score_SE", ILR_vars, first_measurement)
  run_regression("raw_score_GMS", ILR_vars, first_measurement)
  run_regression("scaled_score_GMS", ILR_vars, first_measurement)
  run_regression("zbmi", ILR_vars, first_measurement)
}
```

##### 4 Compositional isotemporal reallocation analysis

```
# install.packages("codaredistlm") # For compositional isotemporal reallocation analysis
library(codaredistlm)

# Proportional reallocation

run_proportional_reallocation_analysis <- function(data, outcome) {
  pred_df <- predict_delta_comps(
    dataf = data,
    y = outcome,
    comps = c("SB_mean", "Sleep_mean", "PA_mean"),
    covars = c("gender_child", "age_child_months"),
    deltas = seq(-60, 60, by = 10) / (data$total_time),
    comparisons = "prop-realloc",
    alpha = 0.05
  )

  print(pred_df)
  plot_delta_comp(pred_df, comp_total = data$total_time, units_lab = "min")
}

# Run reallocation analysis for different outcomes
run_proportional_reallocation_analysis(data, "raw_score_GMS")
run_proportional_reallocation_analysis(data, "zbmi")

# One-v-one reallocation

run_one_v_one_reallocation_analysis <- function(data, outcome) {
  pred_df <- predict_delta_comps(
    dataf = data,
    y = outcome,
    comps = c("SB_mean", "Sleep_mean", "PA_mean"),
    covars = c("gender_child", "age_child_months"),
    deltas = seq(-60, 60, by = 10) / (data$total_time),
    comparisons = "one-v-one",
    alpha = 0.05
  )

  print(pred_df)
  plot_delta_comp(pred_df, comp_total = data$total_time, units_lab = "min")
}

# Run reallocation analysis for different score types
run_one_v_one_reallocation_analysis(data, "raw_score_GMS")
run_one_v_one_reallocation_analysis(data, "zbmi")
```
